# Results from a Theory-Guided Survey to Support Breast Cancer Trial Participation: Barriers, Enablers, and What to Do about them

**DOI:** 10.3390/curroncol28030187

**Published:** 2021-05-26

**Authors:** Jamie C. Brehaut, Kelly Carroll, Jenn Gordon, Justin Presseau, Dawn P. Richards, Dean A. Fergusson, Ian D. Graham, Susan Marlin

**Affiliations:** 1 Clinical Epidemiology Program, Ottawa Hospital Research Institute (OHRI), The Ottawa Hospital, General Campus, 501 Smyth Rd, Ottawa, ON K1H 8L6, Canada; kecarroll@ohri.ca (K.C.); jpresseau@ohri.ca (J.P.); dafergusson@ohri.ca (D.A.F.); igraham@ohri.ca (I.D.G.); 2 School of Epidemiology and Public Health, University of Ottawa, Ottawa, ON K1G 5Z3, Canada; 3 Canadian Breast Cancer Network, 331 Cooper St. Suite 602, Ottawa, ON K2P 0G5, Canada; jgordon@cbcn.ca; 4 Clinical Trials Ontario, 661 University Avenue, Suite 460, MaRS Centre, West Tower, Toronto, ON M5G 1M1, Canada; dawn.richards@ctontario.ca (D.P.R.); susan.marlin@ctontario.ca (S.M.)

**Keywords:** trial participation, recruitment, survey, breast cancer, theory, theoretical domains, barriers and drivers to participation

## Abstract

Background: Ensuring adequate, informed, and timely participation in clinical trials is a multifactorial problem. We have previously developed a systematic, tailorable survey development approach that is informed by theory, can identify barriers and enablers to participation, and can suggest recruitment strategies to address these issues. In this study, we surveyed subscribers to the Canadian Breast Cancer Network (CBCN) in order to identify a comprehensive list of theory-informed barriers and enablers relevant to participation in a hypothetical breast cancer trial. Methods: We developed and conducted an online survey of breast cancer patients informed by the Theoretical Domains Framework and designed to determine previous experience with clinical trials, knowledge about clinical trials, and importance of a comprehensive list of barriers and enablers to trial participation. Participants were contacted by email or through social media. Results: From 2451 subscribers of the CBCN, we received 244 responses and 210 completed surveys (244/2451 or 9.9% participation, 210/244 or 86.1% completion). A total of 38% of respondents indicated experience in trial participation, but 83% indicated confidence in their knowledge about clinical trials. Those who had previously participated in clinical trials were more confident in their knowledge (χ^2^= 6.77, *p* = 0.009) and answered more knowledge questions (t = −3.90 *p* = 0.000). Endorsed barriers and enablers to participation included 39 factors across 12 of 14 domains relevant to behaviour change. Our approach identifies barriers that might be meaningfully addressed by careful knowledge provision (‘*If I would learn more about my condition’; ‘If I find the trial documents hard to understand*’), those that may require other theory-informed approaches to address (‘*my feelings about the quality of my drug plan*’; ‘*my worry over unknown side effects*’), and those that may require tailored approaches depending on participant differences such as previous experience in trials (‘*If there were patient-friendly decision-making tools to help you make your participation decision*’). Discussion: This work demonstrates that a comprehensive, theory-guided survey of barriers and enablers to participation in breast cancer clinical trials is feasible, can lead to detailed knowledge about the issues related to participation in specific trials, and most importantly, can lead to insights about evidence-based ways to better support patient participation.

## 1. Introduction

Ensuring sufficient participation in research is a perennial challenge; participation rates are consistently low for both public and private research enterprises [1,2]. Clinical research is no different; in the U.S., 40% of National Cancer Institute-funded clinical trials have been shown to be discontinued, nearly half because of the inability to obtain enough participants [3]. While cancer trials have been at the forefront of research on improving recruitment [4], it is still the case that only a small proportion of breast cancer patients participate in trials [5]. The harms associated with uncompleted breast cancer studies are substantial, and include wasted resources, opportunity costs, delayed innovation, potentially biased results, potentially reduced public trust, and ethical problems associated with exposing participants to risk without any scientific gain [6].

Participation in breast cancer trials is increasingly seen as a multifactorial problem, spanning patient-specific [7], social [7,8] and systemic issues such as narrow eligibility criteria and poor access to trials [9]. Studies have identified dozens of factors that might affect participation under different circumstances and for different trials [7,8,10,11,12,13,14,15]. We propose that a systematic way is needed to identify and address the factors relevant to specific trials (as opposed to trials in general), and then match these factors with recruitment activities (e.g., study advertisement, informed consent processes, participation support) known to address these factors; in essence, forming a tailored ‘recruitment strategy’ for that specific trial.

To this end, we developed an approach to designing recruitment barrier/enabler surveys that is explicitly informed by theory of human behaviour change. Rather than focusing primarily on awareness of and knowledge about clinical trials (e.g., [16]), the Theoretical Domains Framework (TDF) [17,18,19], which organizes over 100 constructs known to be associated with behaviour and behaviour change into 14 domains, can be used to group recruitment barriers and enablers into categories for which effective change strategies are known [20]. Our development work showed that this approach identifies a wider range of barriers and enablers than existing survey-based approaches, and produces a list of barriers and enablers that is more specific to the individual trial than other survey-based approaches. Furthermore, because the TDF is a core component of a larger literature on how to effect behaviour change in health care [19,21,22], a considerable amount is known about the approaches known to be effective for each of the 14 domains. By identifying relevant barriers/enablers and organizing them by domain, we can leverage the considerable knowledge about how to effect behaviour change in other contexts to tailor study-specific recruitment strategies.

The development process that led to this novel survey approach has been outlined elsewhere [20]. Here, we report the results of a survey of subscribers to the Canadian Breast Cancer Network (CBCN) in order to (1) assess knowledge of and views towards participation in clinical trials; (2) identify the full range of barriers and enablers reported as relevant to participation in a hypothetical breast cancer trial, and (3) provide examples of how framing the barriers and enablers to participation in the context of a theoretical framework like the TDF could be used to design more theory-guided, study-specific recruitment strategies.

## 2. Methods

### 2.1. Reporting and Ethics

This work was reviewed and approved by the Ottawa Hospital Research Network Research Ethics Board (Protocol #20180250). We followed the reporting standards from the CHERRIES guidance for reporting online surveys [23].

### 2.2. Survey Development

This work is part of a larger initiative with Clinical Trials Ontario (CTO), an independent, not-for-profit organization established with support from the provincial government to improve the environment for the conduct of clinical trials in Ontario, Canada. As part of this mandate, CTO prioritized working with various health charity and patient groups to conduct surveys of patients about their knowledge about, attitudes towards, and participation in clinical trials and clinical research. The CBCN was the first of these groups to partner with us to develop a standard, theory-guided approach for identifying barriers and enablers to clinical research participation via online surveys.

A detailed account of the development of this patient focused and theory-guided survey is described elsewhere [20]. To summarize, we used the Theoretical Domains Framework (TDF), which organizes over 100 constructs known to be associated with behaviour and behaviour change into 14 domains that describe determinants of professional and patient health behaviours, to inform the development of an adaptable survey about barriers to and enablers of clinical trial participation. After searching the literature for barriers and enablers to trial recruitment relevant to each of the TDF domains, we used pilot interviews to tailor the survey to identify barriers and enablers to trial participation for CBCN members. Eight think-aloud interviews with patient members of the CBCN ensured the clarity the survey, elicited opinions about which barriers/enablers were relevant to them, and identified additional barriers/enablers. Interviews proceeded iteratively, with changes incorporated into subsequent interviews. Once these interviews were completed and changes incorporated, we created the web-based version of the survey described here.

### 2.3. Survey Content

The anonymous web-based survey consisted of five sections: (1) a welcome page in English and French with an option to complete in either language, (2) Questions about You (demographics and trials experience), (3) Knowledge about Clinical Trials, (4) Barriers and Enablers to Participating in Clinical Trials (spanned over 2 screens), and (5) a Thank-You/Contact Information page. To avoid multiple response problems, there were no back buttons, and responses were saved as soon as the “Next” tab was clicked. Unique site visitors were identified by the session ID, which was created as soon as the respondent clicked “start the survey” link at the bottom of the welcome section. Duplicate database entries with the same session ID were addressed by retaining the most recent data for analysis.

The **Questions about You** section asked whether the respondent or a family member has ever been diagnosed with breast cancer (via drop-down menu: Yes, me; Yes, a family member (with text box to specify relationship), no), stage of breast cancer (drop down menu: early, late, don’t know), time since first diagnosis (text box), have they ever been approached to participate in any kind of research study about breast cancer(yes/no), have they ever actually participated in any research study about breast cancer (clinical trial, survey, interview, database study, don’t know, other), and have they ever looked for a clinical trial to participate in (yes/no). Respondents were also asked whether they received any help in their search, and if a service or navigator would have helped. Demographics collected included postal code, age, gender, education, household income, work status or main activity, ethnic background, number of months of last year residing in Canada, and language first learned at home. Respondents were also asked whether they were confident they could explain what a clinical trial was to a friend or a family member (via drop-down menu: Not at all, Not very, Somewhat, Completely). This section included a total of 17 questions, with some adaptive questioning to reduce the complexity and number of questions asked. For instance, depending the respondent’s selections to various questions (i.e., a family member was diagnosed with breast cancer—questions that followed were phrased “have *they* ever actually participated in any research study”), phrasing was adjusted accordingly, and follow-up questions presented (i.e., responded “yes” to participating in research—a drop down list was presented).

The **Knowledge about Clinical Trials** section included 13 individual items selected from the objective knowledge component of the Quality of Informed Consent (QuIC) instrument [24] and modified to be appropriate for a hypothetical breast cancer trial. Respondents were asked to indicate if they agreed or disagreed with each of the statements. If they were unsure or could not remember, they were asked to select ‘unsure’. Response options presented as radio buttons as in the original scale (Disagree, Unsure, Agree). For analysis, we recoded responses as correct (agreed with correct statement) or incorrect (agreed with incorrect statement, or unsure).

The **Barriers and Enablers to Participation** section included 39 questions in which respondents were asked to imagine they were being asked ‘to participate in a clinical trial investigating how well a new treatment works for breast cancer’. Respondents were asked to rate (using checkboxes) how each issue might affect their decision about whether or not to participate in the clinical trial; specifically, would it push them AWAY from participating (a little, a lot) or push them TOWARDS participating (a little, a lot), or have no effect.

The survey content underwent considerable pilot testing as part of the development process reported elsewhere [20], and web-based elements were also piloted among the team to ensure ease of use, understandability, and functionality of the final form of the survey.

### 2.4. Recruitment and Sample Frame

The CBCN database consisted of 5210 active subscribers; it is updated on a monthly basis and maintained internally by the team at the CBCN. The majority of the subscribers are Canadian breast cancer patients and caregivers; there are also a number of health care professionals and members of community organizations who are a part of this database. This database is primarily used to provide educational information to breast cancer patients and caregivers, including information about the latest research, treatments, programs and events, as well as helping promote Canadian research. CBCN sends out a monthly newsletter in English and French to their subscribers, including information about various research opportunities, events and new educational publications/resources. There are approximately 3–4 emails per month sent to this database.

### 2.5. Survey Administration

The online survey was programmed by and housed at the Ottawa Hospital Methods Centre.

Pre-notifications, mailouts and reminders were administered according to Dillman’s Tailored Design method for surveys [25]. The CBCN sent a prenotification email to individuals on their mailing subscriber list on 21st October 2019. On 23rd October, an initial sample of 20% of subscribers received the official invitation that included a short description of the survey, an estimated time it would take to complete the survey (approximately 15–20 min), contact information, the web survey link, along with the REB approved participant information sheet as an attachment. Participants were told that the survey was anonymous, and participation was voluntary. On 25th October, the remainder of the sample received the invitation email. Two follow-up emails were sent at 1-week intervals to the entire sample; since the survey was anonymous, all follow-ups also went to the full list, regardless of response status. Information about the survey (along with the participant information sheet and survey link) was displayed on the CBCN webpage on 9th December 2019, along with media postings occurring on their Facebook and Twitter pages weekly until 9th January 2020. The survey was taken offline on 27th January 2020 and data downloaded to Microsoft Excel for data cleaning and then to SPSS [26] for analysis. No monetary incentives were provided for completing the survey.

### 2.6. Data Cleaning

Data from participants with the same IP address were retained (2/249), as long as they were not identical, as we wanted to allow multiple patients and caregivers from the same household to participate. Duplicate database entries were identified by examining session IDs and timestamps.

### 2.7. Analysis

Descriptive analyses included frequencies and percentages for all demographic, experience, and knowledge variables, as well as for ratings of the barriers and enablers to trial participation. Means and standard deviations were calculated for continuous variables. A priori tests included an independent-samples t-test to examine the effect of previous trial experience on clinical trial knowledge scores. A post hoc exploratory series of chi-square analyses (uncorrected for alpha) looked at differences in responses to rated barriers/enablers between those who did and did not have experience in research studies.

## 3. Results

**Figure 1:**
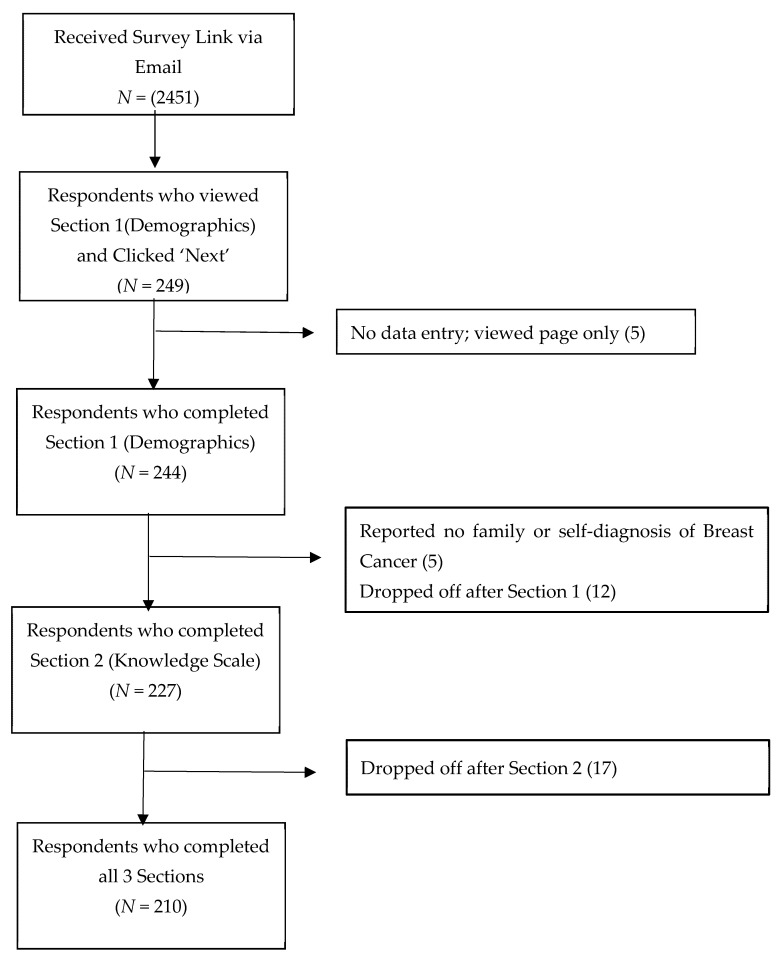
Study Flow Diagram.

Figure 1 describes the response flow diagram for our survey. Contacts were sent via email to 3704 subscribers to the CBCN with active email addresses; 1253 emails bounced back, leaving 2451 emails received. Of those, 249 clicked ‘Next’ at the bottom of the first page of the survey, 244 completed the initial demographics section of the survey, and a total of 210 completed all three sections of the survey (participation rate 244/2451 or 9.9%; completion rate 210/244 or 86.1%). Because we were unable to assess how many emails were opened, and because we could not assess whether people saw the invitation through social media or the website, we were unable to assess view rate (unique survey visitors/unique site visitors). We could not assess response bias because CBCN does not keep personal information about its subscribers.

Table 1 describes self-reported demographic and breast cancer-specific characteristics of our respondents. Most described themselves as female (97%), with a mean age of 57 years (range 31–84). Most respondents lived in Ontario (41%) or Western Canada (28%), with smaller numbers from Eastern Canada (17%) and Quebec (13%). The sample reported high education, with 70% indicating a college or undergraduate degree or higher; reported income was also high, with only 16% reporting household incomes less than $50,000 (although 27% of responses were either missing or prefer not to answer for this question). Respondents overwhelmingly were White (91%), full-time residents of Canada (95%), spoke English at home (73%), and were either retired (38%), working full-time (27%) or on long term disability (15%). A large majority of respondents themselves had experienced breast cancer (92%, or 225/244). Of these, 46% (104/225) described their disease as ‘early stage’, and 40% as ‘late stage’. A total of 6% (14/244) of respondents were family members of people with breast cancer.

Table 2 describes reported experience with and knowledge about clinical trials. When asked whether they had been approached to participate in clinical research, 41% indicated that they had ever been approached for clinical research, with nearly the same number 38% indicating that they had participated in clinical research; the most common type of research included surveys 57%, clinical trials 47%, and database studies 44%. Subjective confidence about whether they could explain what a clinical trial was to a friend was relatively high, with 35% indicating ‘completely confident’, and another 48% indicating ‘somewhat confident’; only 5% indicated that they were ‘not at all confident’ that they could explain what a clinical trial was. Performance on our items assessing knowledge about clinical trials yielded a mean number correct of 9/13 across 227 people completing that section (range 0–13).

Table 3 describes the proportion of 210 respondents rating 38 items from 12 theoretical domains as perceived barriers or enablers to participation ‘in a hypothetical trial of a new treatment for breast cancer’. Our interview-based development process leads to barriers and enablers endorsed by the larger target patient group. Of the 38 items identified through the development process, only 2 failed to be endorsed by a majority of survey respondents. One item, ‘*My worry that participation would mean that others would find out about my condition*’, was endorsed by less than 15% of respondents (8.3% barrier, 6.4% enabler); this item was suggested by the study development group, and occasionally endorsed in our piloting sessions, but not by the broader group. Similarly, ‘*If I had to have more blood tests*’, which received <30% endorsements (20.1% barrier, 7.8% enabler), came up in one interview particularly, but results showed the majority of respondents did not consider it an important issue. The remaining 36 items were endorsed by larger proportions of respondents, supporting their perceived relevance to trial participation decisions.

Of these items, seven of the 38 items identified during our development process fell under the domain of Social Influences. Of these, four were perceived to be enablers towards participation (*regular study updates, physician thinks I should participate, helpful people on hand, family thought I should participate*), one item was perceived to be a barrier (*if physician was paid*), one item showed a difference of opinions on whether it was a barrier or an enabler (*whether trial funders can be trusted*), and one item was rated as having no effect by the majority of respondents (*others would find out about my condition*). Another 7 of the 38 items fell under the domain of Beliefs about Consequences, which focus on issues perceived to be caused by trial participation. Four of these were perceived enablers to participation (*will help me with my condition, will help others, will contribute to science, better care*), two items were rated as barriers (*staying longer in hospital, having more biopsies*), and one item was rated as having no effect by the majority of respondents (*having more blood tests*).

Four of the 38 items focused on perceived Beliefs about Capabilities, which address issues related to whether the patient feels able to participate; 3 were identified as enablers (*cancer prognosis is poor, control over what is happening, health is otherwise good*), and one item showing varied opinions as to whether it was a barrier, a driver, or would have no effect (*quality of drug plan*). Four items focused on methods of Reinforcement to reward participation, three of which were perceived as *enablers (receiving study results, gaining access to new study drugs, reimbursing expenses*), and one item generally perceived as having no effect (*experience with previous trials*). Another four items focused on Goals of the participant and how participation helped meet those goals; three were perceived as barriers (*affecting social life/family commitments, preventing from other activities, interfering with other goals*), and one most commonly perceived as having no effect (*interfering with childcare responsibilities*). Four items also fell under the domain Environmental Context and Resources, which tap into elements of the environment that could affect participation; two were perceived as enablers (*patient-friendly decision-making tools, transportation to/from study appointments*), one perceived as a barrier (*time commitment*), and one item received varied responses (*quality of health care system*).

The remaining items were distributed among the other six domains, including two barriers related to Skills needed for participation, two enablers about the Social Roles involved and how they might affect participation, one enabler focused on the Knowledge to be obtained from participation, one enabler about Optimism around outcomes of participation, one enabler about Memory and reminders during participation, and one barrier about negative Emotions relevant to participation.

We also explored whether knowledge about trials and the barriers/enablers seen as relevant to participation varied according to experience in participating in clinical research. Table 4 shows these comparisons. Respondents with self-reported research experience were more confident in their knowledge about clinical trials (92.3% vs. 79.7%, χ^2^ = 6.77, *p* = 0.009) and answered significantly more of the objective knowledge questions correctly (mean # correct/13: 9.8 vs. 8.8, t = −3.90; *p* = 0.000). Experience with research was also related to differences in perceptions of some barriers and enablers. Post hoc exploratory comparisons also indicate that those with research experience were more likely to see ‘*role as a good citizen*’ as relevant to their participation decision (72.8% vs. 54.8%, χ^2^ = 7.81, *p* = 0.02), those with experience were also more likely to see previous trial experience as an enabler towards participation (46.3% vs. 15.4%, χ^2^ =23.27, *p* = 0.000), and those with experience were less likely to think that patient-friendly decision-making tools would be helpful (76.8% vs. 92.0%, χ^2^ = 11.32, *p* = 0.003).

## 4. Discussion

This work demonstrates that a comprehensive, theory-guided survey of barriers and enablers of participation in breast cancer clinical trials is feasible, can lead to detailed knowledge about the issues related to participation in trials, and most importantly, can lead to insights about evidence-based ways to tailor recruitment strategies. Our survey development process produced 38 items specific to potential participants in breast cancer trials based on 14 theoretical domains of behaviour change. We have previously demonstrated that survey instruments focused on identifying barriers and enablers to participation typically cover only a few of the theoretical domains proposed by the TDF, and efforts to encourage participation in trials have predominantly focused on changing the information that is provided to potential participants [20]. In contrast, endorsed items from this survey spanned 12 of 14 TDF domains. We propose that by explicitly considering these theoretical domains during trial development, we will identify more barriers and enablers to participation in a trial, and thus better understand the full range of challenges to its successful completion. Furthermore, because each TDF domain is informed by a theoretical literature describing relevant mechanisms underlying behaviour change, knowledge about which domains are ‘in play’ provide clearer suggestions about how to tailor recruitment strategies.

Efforts to tailor recruitment strategies based on these results could take at least two distinct approaches. One might be to address the individual issue by incorporating it into the overall recruitment strategy. For example, nearly 90% of respondents indicated that ‘*learning more about their condition*’ would be seen as an enabler of participation; communicating and ensuring this as a potential benefit of trial participation might therefore contribute to increased participation and more satisfied participants. Similarly, over 80% of respondents indicated that ‘*worry over unknown side effects*’ would be a barrier to participation in a breast cancer trial; providing clear information about side effects and their likelihood might also increase recruitment and trial experience. Note, however, that while this approach essentially provides testable hypotheses, it does not help to implement these changes effectively, provide a theoretical justification for what should work when, or give guidance on the potential generalizability of such optimization strategies.

Our theory-informed development approach also suggests a second, more theoretically supported, generalizable, and nuanced approach to tailoring recruitment strategies. Based on the literature on behaviour change [21,27], we can identify and implement change strategies known to be effective when barriers within specific theoretical domains are involved. For example, the ‘*worry over unknown side effects*’ barrier discussed earlier falls within the Emotions domain. Based on behavioural theory supporting the TDF, strategies known to address issues related to emotional barriers include engaging in stress management (e.g., alleviating worry by underscoring the quality of care that will be received, risk mitigation strategies in place), and coping planning (e.g., helping patients design ‘if-then’ plans to engage in if they feel they are experiencing an unknown side effect). Furthermore, this theory-informed approach may help identify interventions unlikely to be successful. For example, in a situation where emotional issues are a primary barrier to trial participation, behavioural theory tells us that interventions such as providing more reminders, changing the environment to encourage participation (e.g., more study posters), and additional information provision about the trial (e.g., adding information to the consent form) are unlikely to benefit the participant. This second approach can therefore help us tailor recruitment strategies to the specific challenges faced by participants in the specific study, and do so in a way that is both more likely to be effective and less likely to waste effort.

Note that the strength of this theory-informed approach is not to create a single index of generic barriers that can be applied across many trials and clinical situations. Instead, it enables trial-specific surveys that are informed by a common comprehensive, theory-guided framework. Whether by starting with an existing list of barriers/enablers and adding/deleting relevant ones based on patient consultation, or using a TDF-focused interview guide to identify barriers as described here, we propose that using this framework-guided approach can have several benefits. First, it has the potential for a much broader utility than any individual scale that might be modified or discarded because some individual items are inappropriate. Second, because the approach involves a framework that is designed to facilitate implementation, there is potential to provide guidance based on existing literature about how most effectively to intervene with barriers stemming from specific domains [21]. Finally, the approach can facilitate the science of recruitment by linking recruitment results to the likely mechanisms leading to the results, as opposed to focusing on categories of intervention (e.g., modifying information, changes in trial conduct) that do little to inform when and where a certain type of mechanism is likely to work.

Our results point to an important issue about the variance with which different people can perceive items as barriers or enablers. In contrast to most survey scales that frame issues in a unipolar fashion, it became clear during our development process that some items could be perceived both as a barrier or an enabler depending on patient views. For example, while many respondents perceived ‘*my health is good (other than my cancer)*’ as an enabler towards trial participation, some interviewees rated this issue as a barrier to participation, perhaps reasoning that better health should imply prioritizing individual life pursuits over trial participation. This informed our two-sided response scale for the survey. Items that were perceived as a barrier or an enabler depending on the participant included ‘*my health is good (other than my cancer)*’ (15% rated as a barrier, 59% as an enabler), ‘*my feelings about the quality of my drug plan*’ (20% barrier, 24.8% enabler); ‘*My feelings about the quality of the health care system*’ (20.1% barrier; 29.4% enabler); ‘*My feelings about whether the trial funders can be trusted*’(26.5% barrier; 50.9% enabler); and ‘*If the consent documents describe probabilities of side effects and numbers of patients affected by them*’ (44.5% barrier, 25.7% enabler). Existing scales do not address this issue, but such items may be particularly relevant when considering how to tailor recruitment strategies (e.g., by assessing opinions on these issues and changing the recruitment strategy accordingly).

Our findings also showed that the barriers and enablers endorsed by respondents differed according to level of experience with clinical trials. Those with previous experience were more likely to rate participation as part of their ‘*role as a good citizen*’ and their ‘*previous trial experience*’ as relevant to whether they would participate in the new hypothetical trial, but were less likely to rate ‘*patient-friendly decision-making tools*’ as relevant to their participation. Because the analyses were post hoc, we cannot assess with any certainty the reasons contributing to these differences, but the notion that our approach is sensitive to differences in respondent experience could lead to further ways to tailor recruitment strategies.

### Limitations

Our study has a number of limitations that warrant consideration. Several challenges to the representativeness and generalizability of our results require that this study be replicated in other contexts and populations. First, our sampling frame (subscribers to the CBCN) disproportionately includes members that are white and well educated, and so likely does not reflect the full population of all Canadian breast cancer patients. Similarly, given our overall low response rate (9.9% of all CBCN members), our sample likely is non-representative of some aspects of the population. While detailed demographic characteristics of subscribers to the CBCN were not available to us, our sample of respondents consisted largely of white, well-educated, well-informed, English-speaking women. Statements about generalizability of these results must therefore be made with care. We note that with approximately equal numbers describing their disease as early vs. later stage, and only a minority reporting any experience with clinical research, our sample did vary usefully in terms of these factors.

We asked respondents to answer survey questions in light of a generic clinical trial for breast cancer, which likely affected the barriers, enablers, and TDF domains represented in the current survey. For example, we previously noted that one TDF domain (Intention) did not end up being included in the survey at least in part because interviewees found it difficult to think about whether they ‘intended’ to participate in a generic, hypothetical trial [20]. In contrast to this generic application of our theory-based survey development approach, we see the chief utility of the approach as a way to help trialists identify factors likely to affect participation *in their specific trial*, by surveying potential participants in advance of trial onset. Implementing this approach for specific trials will allow us to assess whether specific descriptions of the trial will lead to more specific barriers/enablers being identified, more tailored recruitment strategies to be developed, and ultimately more successful recruitment.

This work shows that a theory-informed approach to barriers/enablers in survey development can help identify a more comprehensive range of potential issues related to the ultimate success of clinical trial recruitment. Furthermore, because the approach implicates theoretical domains for which effective change strategies are known, it can provide useful guidance for trialists seeking to tailor their recruitment strategies in the most effective and resource-efficient ways possible.

## Figures and Tables

**Table 1:** Self-reported demographics and breast cancer-specific characteristics (*N* = 244).

		*N*	Percent
Gender	Male	2	0.8%
	Female	238	97.5%
	Transgender/non-binary	0	0%
	Prefer not to answer	0	0%
	Missing	4	1.6%
Age		Range (31–84)	Mean (SD) 57.0 (11.5)
Geographic location	Ontario	100	41.0%
	Western Canada	68	27.9%
	Eastern Canada	41	16.8%
	Quebec/Northern Canada	33	13.5%
	Missing	2	0.8%
Education	Some high school	4	1.6%
	High school diploma	13	5.3%
	Some university/college	42	17.2%
	College diploma/BA degree	122	50.0%
	Graduate degree	30	12.3%
	Doctoral degree	8	3.3%
	Professional degree	12	4.8%
	Other	2	0.8%
	Prefer not to answer	2	0.8%
	Missing	9	3.7%
Household income	Less than $50,000	40	16.4%
	$50,000 or more but less than $100,000	81	33.2%
	$100,000 or more but less than $150,000	26	10.7%
	$150,000 or more	30	12.3%
	Prefer not to answer	52	21.3%
	Missing	15	6.1%
Ethnicity	White/Caucasian	223	91.4%
	Asian	3	1.2%
	South Asian	3	1.2%
	Black	2	0.8%
	Arab/West Asian	0	0%
	First Nations/Indigenous	4	1.6%
	Filipino	0	0%
	Latin American	1	0.4%
	Other (e.g., participant reported)	5	2.0%
	Prefer not to answer	3	1.2%
Months in Canada previous year	9 Plus months	233	95.5%
	6–9 months	3	1.2%
	Less than 3 months	2	0.8%
	Prefer not to answer	3	1.2%
	Missing	3	1.2%
Language spoken at home	English	179	73.4%
	French	41	16.8%
	Other	19	7.8%
	Missing	5	2.0%
Employment	Retired	94	38.5%
	Full-time employment	66	27.0%
	Long-term disability	37	15.2%
	Self employed	20	8.2%
	Part-time employment	19	7.8%
	Other	24	9.8%
	Prefer not to answer	6	2.5%
Respondents with breast cancer		225	92.2%
Early stage		104	46.2%
Late stage		91	40.4%
Don’t know		22	9.8%
Missing/no response		8	3.6%
Respondents were family member with breast cancer		14	5.7%

**Table 2:** Reported experience with and knowledge about clinical trials (*N* = 239).

Question	*N* (% Total)
Responded ‘no’ to being approached for research	129 (54.0%)
Responded ‘yes’ to being approached for research	97 (40.6%)
Responded ‘no’ to ever participating in research	134 (56.1%)
Responded ‘yes’ to ever participating in research	91 (38.1%)
What did participation involve?	
Clinical trial	43 (47.3%)
Survey	52 (57.1%)
Interview	20 (22.0%)
Database study	40 (44.0%)
Don’t know	0 (0.0%)
Other	12 (13.2%)
Confidence in clinical trial knowledge?	
Not at all confident	13 (5.4%)
Not very confident	24 (10.0%)
Somewhat confident	115 (48.1%)
Completely confident	83 (34.7%)
Missing	4 (1.7%)
Responded ‘yes’ to having actively looked for a clinical trial	65 (27.2%)
Searched online	57 (87.7%)
Asked a health care provider	37(56.9%)
Spoke to other patients	16 (24.6%)
Other	5 (7.7%)
Responded ‘yes’ to having help in search	16 (24.6%)
Help from doctor	6 (37.5%)
Help from other patients	2 (12.5%)
Other help	7 (43.8%)
Missing	1 (6.3%)
Responded ‘yes’ to finding a study to participate in	13 (20.0%)
Responded ‘yes’ to desire for search navigator	53 (81.5%)

**Table 3:** Barriers and enablers to participation in a hypothetical trial of a new treatment for breast cancer (*N* = 210) *N* (%).

Statement	Perceived as Barrier			Perceived as Enabler	
	A Lot	A Little	No Effect	A Little	A Lot
Social Influences					
If the investigators provided regular study updates	1 (0.5%)	0 (0%)	10 (4.9%)	89 (43.2%)	106 (51.5%)
If my physician (s) thought I should participate	0 (0%)	0 (0%)	13 (6.3%)	76 (37.1%)	116 (56.6%)
If there were helpful people on hand to help you make your participation decision	0 (0%)	2 (1.0%)	17 (8.2%)	96 (46.4%)	92 (44.4%)
If my family thought I should participate	0 (0%)	1 (0.5%)	39 (19.1)	100 (49.0%)	64 (31.4%)
If my physician was paid to recruit patients into the study	122 (59.5%)	41 (20.0%)	38 (18.5%)	3 (1.5%)	1 (0.5%)
My feelings about whether the trial funders can be trusted	23 (11.3%)	31 (15.2%)	46 (22.5%)	46 (22.5%)	58 (28.4%)
My worry that participation would mean that others would find out about my condition	8 (3.9%)	9 (4.4%)	175 (85.4%)	9 (4.4%)	4 (2.0%)
Belief about Consequences					
My hope that participation will help me with my condition	0 (0%)	0 (0%)	3 (1.5%)	33 (16.1%)	169 (82.4%)
My belief that participating would help others	0 (0%)	0 (0%)	5 (2.5%)	85 (41.9%)	113 (55.7%)
My belief that participating would contribute to science	0 (0%)	0 (0%)	13 (6.3%)	104 (50.0%)	91 (43.8%)
My belief that I would receive better care if I participated	1 (0.5%)	0 (0%)	31 (14.9%)	101 (48.6%)	75 (36.1%)
If I had to stay longer in hospital	40 (19.5%)	78 (38.0%)	78 (38.0%)	5 (2.4%)	4 (2.0%)
If I had to have more biopsies	28 (13.8%)	63 (31.0%)	99 (48.8%)	5 (2.5%)	8 (3.9%)
If I had to have more blood tests	9 (4.4%)	32 (15.7%)	147 (72.1%)	9 (4.4%)	7 (3.4%)
Belief about Capabilities					
If I think my cancer prognosis is poor	13 (6.2%)	6 (2.9%)	17 (8.1%)	52 (24.9%)	121 (57.9%)
My belief that participating would give me a sense of control over what is happening to me	0 (0%)	4 (2.0%)	28 (13.7%)	86 (42.0%)	87 (42.4%)
If I think my health is good (other than my cancer)	15 (7.2%)	16 (7.7%)	55 (26.3%)	74 (35.4%)	49 (23.4%)
My feelings about the quality of my drug plan	12 (5.9%)	29 (14.1%)	112 (54.6%)	30 (14.6%)	21 (10.2%)
Reinforcement					
If I received the results of the study once it was complete	1 (0.5%)	3 (1.5%)	14 (6.9%)	84 (41.4%)	101 (49.8%)
If I would gain access to new study drugs	1 (0.5%)	4 (1.9%)	17 (8.3%)	77 (37.7%)	105 (51.5%)
If the study reimbursed expenses	1 (0.5%)	0 (0%)	38 (18.2%)	94 (45.0%)	76 (36.4%)
My experience with previous trials	2 (1.0%)	9 (4.4%)	138 (67.3%)	27 (13.2%)	29 (14.1%)
Goals					
If I think participation would affect my social life/family commitments	37 (18.0%)	106 (51.7%)	58 (28.3%)	2 (1.0%)	2 (1.0%)
My belief that participation would prevent me from my other activities	39 (18.7%)	98 (46.9%)	65 (31.1%)	4 (1.9%)	3 (1.4%)
My belief that participation would interfere with other goals of mine	36 (17.6%)	102 (49.0%)	64 (31.4%)	2 (1.0%)	2 (1.0%)
If I think participation would interfere with my childcare responsibilities	33 (16.1%)	42 (20.5%)	129 (62.9%)	1 (0.5%)	0 (0%)
Environmental Context and Resources					
If there were patient-friendly decision-making tools to help you make your participation decision	0 (0%)	1 (0.5%)	28 (13.4%)	107 (51.2%)	73 (34.9%)
If the study provided transportation to/from study appointments	0 (0%)	1 (0.5%)	75 (35.9%)	63 (30.1%)	70 (33.5%)
If I think there is a substantial time commitment	31 (14.9%)	79 (38.0%)	80 (38.5%)	11 (5.3%)	7 (3.4%)
My feelings about the quality of the health care system	11 (5.4%)	30 (14.7%)	103 (50.5%)	43 (21.1%)	17 (8.3%)
Skills					
If I find the trial documents hard to understand	64 (30.8%)	87 (41.8%)	48 (23.1%)	6 (2.9%)	3 (1.4%)
If the consent documents describe probabilities of side effects and numbers of patients affected by them	17 (8.1%)	76 (36.4)	62 (29.7%)	36 (17.2%)	18 (8.6%)
Social/Professional Role and Identity					
My belief that participating would give me a sense of purpose	1 (0.5%)	1 (0.5%)	48 (23.1%)	96 (46.2%)	62 (29.8%)
My belief that participation is part of my role as a good citizen	8 (3.9%)	4 (1.9%)	68 (32.9%)	77 (37.2%)	50 (24.2%)
Knowledge					
My belief that I’d learn more about my condition if I participated	0 (0%)	1 (0.5%)	24 (11.5%)	104 (50.0%)	79 (38.0%)
Optimism					
My hope that participation would help find a cure	0 (0.0%)	1 (0.5%)	4 (2.0%)	54 (26.3%)	146 (71.2%)
Memory, Attention and Decision Processes					
If the investigators provided telephone reminders about study appointments	1 (0.5%)	3 (1.4%)	100 (48.1%)	71 (34.1%)	33 (15.9%)
Emotion					
My worry about unknown side effects	50 (24.0%)	119 (57.2%)	30 (14.4%)	7 (3.4%)	2 (1.0%)

red = barrier, green = enabler, yellow = no effect.

**Table 4:** Differences in confidence and knowledge about clinical trials and reported barriers/enablers to participation for those with and without research experience.

	Reported Research Experience		Comparison (*p* Value)
	Yes	No	
Confidence in clinical trial knowledge, *N* = 234	91	143	χ^2^ (1) = 6.77 (*p* = 0.009)
Yes	84 (92.3%)	114 (79.7%)	
No	7 (7.7%)	29 (20.3%)	
Knowledge about clinical trials score, M (SD)	9.8 (1.5)	8.8 (2.0)	t (223) = −3.90 (*p* = 0.000)
My belief that participation is part of my role as a good citizen, *N* = 205	81	124	χ^2^ (2) = 7.81 (*p* = 0.02)
Enabler to trial participation	59 (72.8%)	68 (54.8%)	
Barrier to trial participation	5 (6.2%)	7 (5.6%)	
No effect	17 (21.0%)	49 (39.5%)	
If there was patient-friendly decision-making tools to help you make your participation decision, *N* = 207	82	125	χ^2^ (2) = 11.32 (*p* = 0.003)
Enabler to trial participation	63 (76.8%)	115 (92.0%)	
Barrier to trial participation	0 (0%)	1 (0.8%)	
No effect	19 (23.2%)	9 (7.2%)	
My experience with previous trials, *N* = 203	80	123	χ^2^ (2) = 23.27 (*p* = 0.000)
Enabler to trial participation	37 (46.3%)	19 (15.4%)	
Barrier to trial participation	4 (5.0%)	7 (5.7%)	
No effect	39 (48.8%)	97 (78.9%)	

## Data Availability

The datasets used and/or analysed during the current study are available from the corresponding author on reasonable request.

## Abbreviations

TDF	Theoretical Domains Framework
CBCN	Canadian Breast Cancer Network
CTO	Clinical Trials Ontario
QuIC	Quality of Informed Consent
